# Relationship between Enhanced Intensity of Contrast Enhanced Ultrasound and Microvessel Density of Aortic Atherosclerostic Plaque in Rabbit Model

**DOI:** 10.1371/journal.pone.0092445

**Published:** 2014-04-08

**Authors:** Xiangdong You, Pintong Huang, Chao Zhang, Minghui Wang, Ying Zhang, Yurong Hong, Shumei Wei, Chunmei Liu, Zhaoxia Pu, Jianmin Zhang, Shuyuan Chen

**Affiliations:** 1 Department of Ultrasonography, the Second Affiliated Hospital of Zhejiang University School of Medicine, Zhejiang, China; 2 Department of Pathology, the Second Affiliated Hospital of Zhejiang University School of Medicine, Zhejiang, China; 3 Department of Neurological Surgery, the Second Affiliated Hospital of Zhejiang University School of Medicine, Zhejiang, China; 4 Baylor Research Institute, Baylor University Medical Center at Dallas, Dallas, Texas, United States of America; Innsbruck Medical University, Austria

## Abstract

The aim of this study was to evaluate the relationship between enhanced intensity of contrast enhanced ultrasound and microvessel density of aortic atherosclerotic plaque in rabbit model. The abdominal aortas of thirty-six male New Zealand rabbits were damaged by balloon expansion and the animals were then fed a high fat diet for 12 weeks. Twenty-seven plaques on the near aortic wall were detected using conventional ultrasound examination. The maximum thickness of each plaque was recorded. CEUS was performed on these 27 plaques and the time-intensity curves (TICs) were analyzed offline. Using the quantitative ACQ software, features such as the arrival time (AT), time to peak (TTP), baseline intensity (BI), peak intensity (PI) and enhanced intensity (EI) (EI = PI-BI) were assessed. Inter- and intra-observer agreement of EI were assessed using the Bland-Altman test. After CEUS examination, the rabbits were sacrificed for pathological examination and CD34 monoclonal antibody immunohistochemical detection. Microvessel density (MVD) was counted under the microscope. The relationship between indexes of CEUS and the level of MVD was analyzed. There was a good positive linear correlation between EI and MVD (γ = 0. 854, P<0. 001), the intraclass correlations for inter- and intra-observer agreement for EI were 0.73 and 0.82 respectively, suggesting that EI may be act as a useful index for plaque risk stratification in animal models.

## Introduction

Atherosclerosis is a serious public health problem that accounts for more than half of deaths in developed countries. The coronary and carotid arteries, aorta and other peripheral arteries are most commonly involved, and the most common clinical sequelae include sudden cardiac death, nonfatal myocardial infarction, and stroke. The development of vulnerable plaques, which are more likely to rupture and cause symptoms or clinical events, is part of the atheromatous process [1], [2]. Thus, the identification of plaques at risk of rupture is of great clinical importance. In animal models and human pathological studies, neovascularization often precedes the development of atherosclerotic plaques and is an important feature of vulnerable plaques [3]–[5]. Experimental study has demonstrated that angiogenesis inhibitors can reduce plaque growth [6]. In recent years, it has been reported that contrast enhanced ultrasound (CEUS) can be used as a novel, non-invasive tool for detecting intraplaque neovascularization [7]–[9], yet the assessment of contrast enhancement by using visual interpretation is limited because of its subjective nature. Our study was undertaken to assess the relationship between enhanced intensity (EI) of CEUS and microvessel density (MVD) of aortic atherosclerotic plaque in rabbit model.

## Materials and Methods

### Experimental animals

Thirty-six standard 4 month old male New Zealand rabbits, weighing 1.8–3.0 kg were obtained from the Animal Husbandry and Veterinary Research Institute, Zhejiang Academy of Agricultural Sciences. Animals were fed with 150 g/d of an atherogenic diet containing 1.5% cholesterol for 12 weeks after balloon-induced aortic endothelial injury, scanned with CEUS and sacrificed at the end of 12 weeks. All procedures and care of animals were approved by the Animal Management Rules of the Chinese Ministry of Health (document No. 55, 2010), and the Animal Care Committee of Zhejiang University approved the study.

### Apparatus and contrast agent

Ultrasound scanning was performed with an Acuson/Siemens Sequoia 512 apparatus, with standard vascular presets, and equipped with Cadence™ contrast pulse sequencing technology (CPS) [10], which uses phase and amplitude modulation to separate the microbubble signals from tissue echoes, at 7 MHz with a low mechanical index (0.35). A linear phased array probe 15L8 (frequency: 8–14 MHz) was used. The contrast agent SonoVue (Bracco SpA, Milan, Italy) used in this study was supplied as a lyophilized powder, which was reconstituted by adding 5 ml of 0.9% saline and gently shaking the vial by hand to form a homogeneous microbubble suspension. The suspension contains 8 µl/ml sulfur hexafluoride (SF6) gas stabilized by a phospholipid shell (microbubble concentration 5 mg/ml). The mean microbubble diameter is 2.5 µm with an osmotic pressure of 287 mOs/kg.

### Preparation of the model

All rabbits were anesthetized with 4% sodium pentobarbital (40 mg/kg) intramuscularly. The left femoral artery was exposed with the distal end of the free artery ligated and the proximal end clipped using vascular clips. After puncture with a 20G needle under ultrasound guidance, a size 2 Fogarty balloon catheter was passed into the abdominal aorta for about 20 cm. The balloon was then inflated with 2 mL of saline, and the catheter was gently retracted to the common iliac artery. This process was performed 5 times in each rabbit and the catheter was then removed, the proximal end of the femoral artery tied off and the incision closed with sutures. Antibiotics were given to prevent postoperative infection. All the rabbits were then fed an atherogenic diet (containing 1.5% cholesterol, 150 g/d) for 12 weeks.

### Ultrasound examination and measurement of indexes

**Figure 1:**
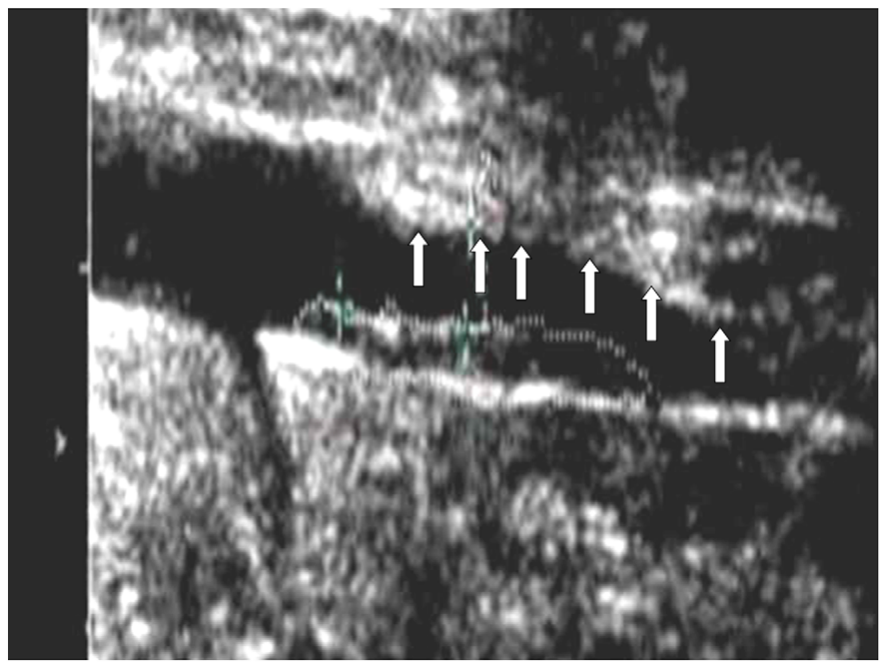
Rabbit abdominal aorta atherosclerotic plaque on the near aortic wall (arrows) was displayed clearly by ultrasonography.

The abdominal aorta of each rabbit was examined using an Acuson 512 imaging system (Siemens, Mountain view, CA) equipped with a 15L8 linear probe. The thickest plaque on the anterior aortic wall in each rabbit was selected and the plaque thickness and area were measured with electronic calipers. The position of the selected plaque was confirmed for later pathological examination by measuring the distance of plaque to the left renal artery. When a clear two-dimensional image was obtained (Figure 1), the image was magnified using the write zoom capability of the scanner and then we selected the CPS-SMALL PARTS preset. Probe output power was −15–−21 dB and the focus position was set at the level of the abdominal aorta. All these settings were kept constant throughout each examination. After shaking, the contrast agent was administered rapidly as 0.1 mL/kg bolus through the rabbit ear vein using a 21G needle. A real time contrast-enhanced abdominal aorta cine-loop (at least 2 minutes long) following injection of contrast material was acquired and digitally stored for off-line analysis.

**Figure 2:**
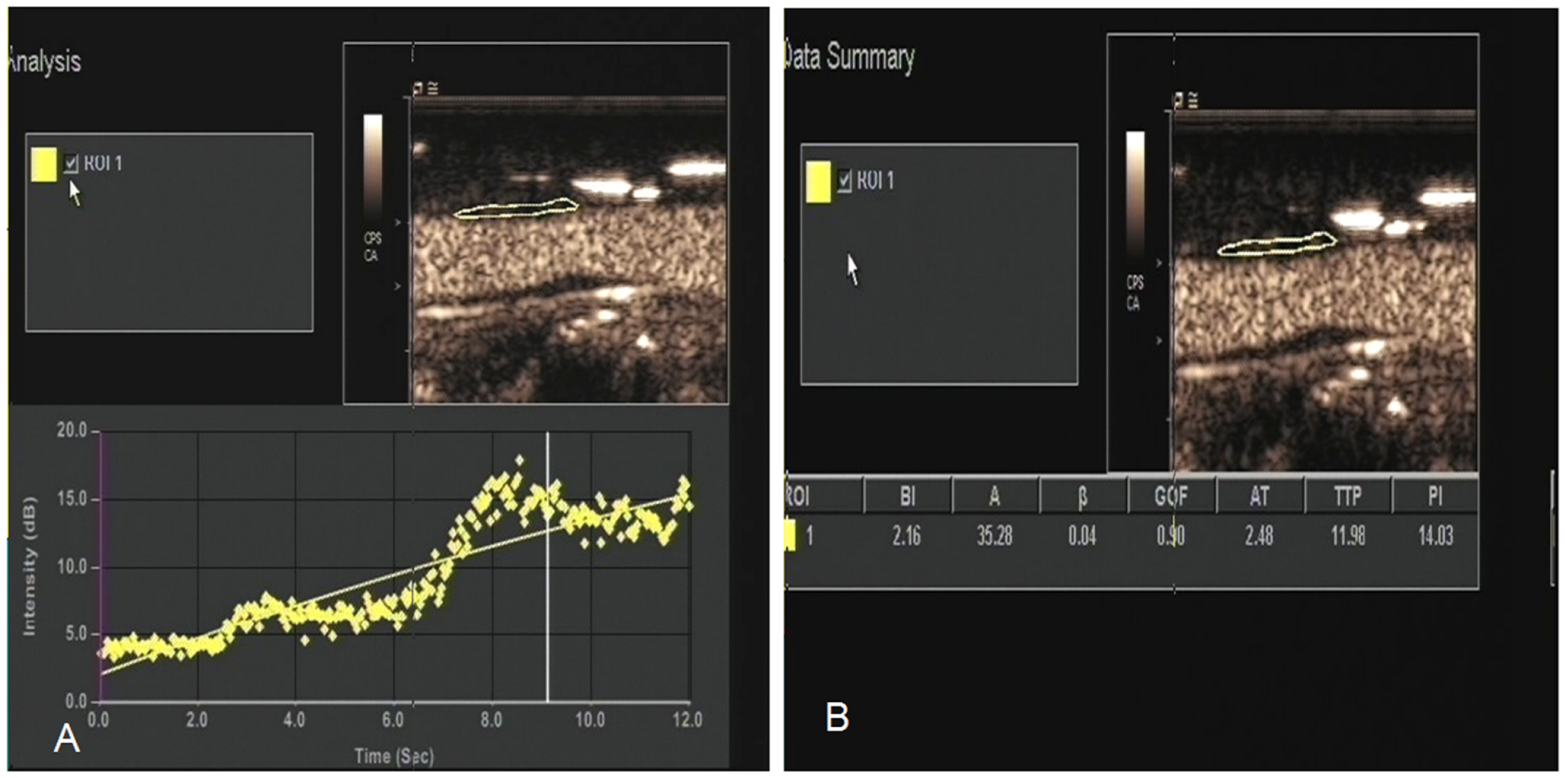
A ROI was drawn freehand around the margin of the plaque. A time-intensity curve (TIC) for the selected tissue was derived automatically by the scanner software (A) and quantification indexes were calculated by the ACQ software (B).

Two board-certified abdominal radiologists (CZ and YZ, with 13 and 11 years of experience) reviewed the cine loops of all the plaques off-line using auto-tracking contrast quantification (ACQ) technology analysis software. A freehand region of interest (ROI) was drawn around the margin of the plaque using an electronic-cursor, avoiding the lumen and surrounding tissue. The ROI was adjusted manually frame by frame as necessary, and a goodness-of-fit above 0.75 was considered acceptable (this described the discrepancy between observed and expected values under the model of logarithmic compression used by the ACQ software). A time-intensity curve (TIC) for the selected ROI was derived automatically by the scanner software and four perfusion indices were calculated by the ACQ software. These were the arrival time (AT) (when the signal is greater than the threshold of 120% of the baseline signal), time-to-peak (TTP), baseline (BI) and peak intensities (PI) [10] (Figure 2). All measurements were performed three times, and the means of these three measurements were calculated and compared. Enhanced intensity (EI) was defined as PI minus BI. The interobserver variability of EI was determined by two radiologists (CZ and YZ) who were blinded to each other's results. The intra-observer variability of EI was evaluated by one radiologist (CZ) who was blinded to the results of the first measurement at two time-points three months apart.

### Immunohistochemistry

**Figure 3:**
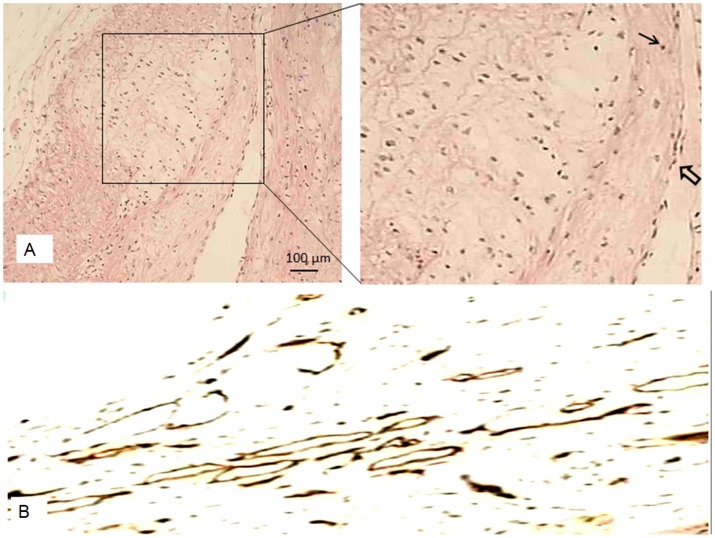
Pathological images of atherosclerotic plaque. (A) Low-power view of a plaque showing a thick fibrous cap (open arrow). The rectangular part was zoomed for clarity. Few inflammatory cells were observed. No necrotic core was found. (B) CD34-stained tissue specimen, with brown microvessel endothelial cells within the plaque (×200).

All rabbits were euthanized immediately after the scans by the intravenous injection of pentobarbitone sodium. Based on the ultrasound landmark of the plaques, the plaques were excised and the specimens were fixed in 10% formalin, paraffin-embedded and sliced into 4 µm-thick serial sections for microscopic examination after staining with hematoxylin & eosin (H&E), and examined for morphological changes. The plaque specimens were also stained with CD34 antibodies, an immuno-histochemical marker. The numbers of microvessels were counted at a magnification of 200× using the method described by Weidner et al [11]. Endothelial cells that stained brown were regarded as CD34-positive (Figure 3). For the microvessel counts, any brown-stained cell or cluster of endothelial cells, clearly separate from tissue cells and other connective tissue elements, was considered as a single vessel. Vessels were counted in the three highest density areas at 200× magnification (using a combination of 20× objective and 10× ocular, 0.74 mm^2^ per field). Microvessel count was expressed as the mean number of vessels in these areas.

### Statistical analysis

Statistical analysis was performed with SPSS 13.0 software. The mean differences and 95% limits of agreement for each of the features of EI for each observer and for both observers were calculated using the Bland-Altman test [12]. Pearson linear correlation analysis was used to study the degree of linear relationship between MVD and the quantitative indexes of contrast enhanced ultrasound imaging.

## Results

No plaque developed in four of the animals. Five plaques on the posterior wall of the aorta in five rabbits were excluded. The remaining 27 rabbits with a total of 27 plaques on the anterior aorta arterial wall were studied with contrast enhanced ultrasound imaging.

**Figure 4:**
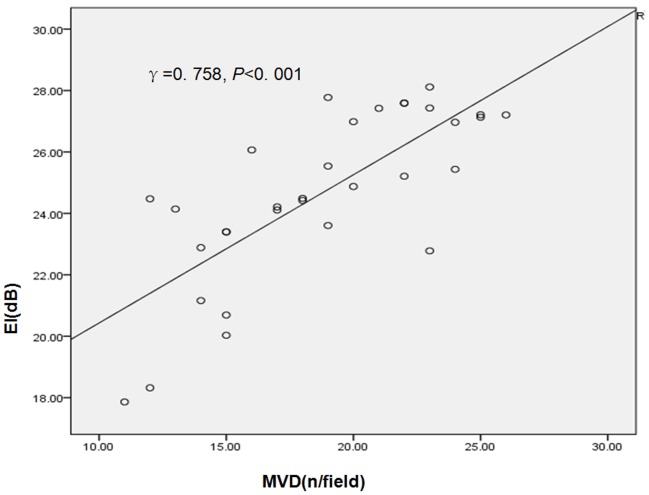
The MVD and EI were correlated in a positive linear manner (γ = 0.854, P<0. 001).

The MVD and EI were correlated in a positive linear manner (γ = 0. 854, P<0. 001) (Figure 4), while there were no correlations between MVD and AT or TTP (γ = 0.106 and −0.135, respectively, P>0. 05 for both).

### Intra- and Inter-observer Agreement

Mean difference, SD, and 95% limits of agreement for intra- and inter-observer measurements for EI were shown in Figure S1 and Figure S2 respectively, with the corresponding scatter and Bland-Altman agreement plots. The intraclass correlation for inter- and intra-observer agreement was 0.73 and 0.82 respectively.

## Discussion

Plaque neovascularization has been well established and confirmed in histologic studies as a consistent feature of vulnerable plaque in patients with cerebrovascular disease [4], [13], [14]. Accordingly, there is a compelling need to develop a non-invasive, cost-effective method for the detection of neovascularization within atherosclerotic plaque to improve carotid plaque risk stratification and assess the response to anti-atherosclerotic therapies. Unlike most contrast agents used for computed tomography and magnetic resonance imaging, the microbubbles used as sonographic contrast-agent are pure intravascular tracers, so that they can only reach plaque tissue by vascular channels; thus microbubbles visualized within the plaque are markers of local neovascularization [15]. Vicenzini et al [16] described the visualization of contrast-agent microbubbles within carotid plaques as a marker of vascularization, though no systematic histological validation was performed. Some experts also reported diffuse contrast uptake in plaques in symptomatic patients [17], [18].

Although previous studies reported that an increase in neovascularization in patients demonstrates extensive enhancement of plaques [19] and carotid plaque contrast-agent enhancement with sonographic agents correlates with histological density of neovessels [20]. The present study showed that only the feature EI was significantly correlated with MVD (γ = 0.854, P<0. 001), indicating its potential value as a new marker of the neovascularization within atherosclerostic plaques in animal model.

The PI and BI were not analyzed in this study because PI measures the maximum intensity of enhancement, and this depends on the baseline intensity (BI), which may differ among plaques, while the change in intensity after enhancement would be expected to be more robust since it is normalized by the BI. EI represents the total number of microbubbles reaching the microvascular bed, and so is related to blood flow; thus the EI reflects the MVD within the plaque. Our study revealed that the reproducibility of EI measurements obtained by the ACQ analysis software to quantify the TICs of the CPS signals from the microbubbles is acceptable for the evaluation of rabbit aorta plaque. The intraclass correlations of inter- and intra-observer were 0.73 and 0.82 respectively, indicating good agreement.

Our study also found no correlations between MVD and AT or TTP (γ = 0.106 and −0.135, respectively, P>0. 05 for both). The main reason could be that these temporal features reflect the process of contrast agent flowing into the lesion, which can be affected by factors such as the speed of the bolus injection, cardiovascular status and so on [21]. Therefore, it is not surprising that they were not well correlated with the MVD.

SC Lee et al. reported that CEUS with maximum intensity projection (MIP) processing can provide quantitative data on temporal changes in the functional density of the vasa vasorum, and they suggested that MIP processing of contrast ultrasound data is the best way to evaluate microvascular density, but they did not create focal atherosclerotic plaque [22]. Hence the ability of quantitative indexes of CEUS to distinguish between vulnerable and stable plaques is still not fully understood. Previous studies reported that plaque stability is related to angiogenesis within the plaque [19], [23]. These microvessels are immature and fragile and thus prone to rupture and hemorrhage, which likely explains plaque instability [24].

A recent study reported that quantification features derived from TICs can be affected by plaque motion. The new motion compensation was used to prevent the plaque ROI from including parts of saturation artifacts and the lumen and minimized the risk of false peak intensities [25]. In this study, we used CEUS indexes obtained by analysis of TICs to evaluate the vascularity of the plaques. The ROI was adjusted manually frame-by-frame from CEUS imaging, and a goodness-of-fit above 0.75 was considered acceptable, which describes the discrepancy between observed and expected values under the model of logarithmic compression used by ACQ software.

In summary, the quantitative index of contrast enhanced ultrasound imaging EI has a good correlation with MVD, which reflects neovascularization within the plaque, and indicating the EI of CEUS may be act as a useful index for plaque risk stratification in animal models.

### Limitations

The optimal dose of contrast agent and mechanical index were not tested in our study. Although biochemical markers such as matrix metalloproteinases, collagen content, etc were not included in the criteria of plaque classification we used, the relationship between EI and these biochemical markers within the plaque needs to be further investigated. We did not perform longitudinal contrast studies to evaluate the time course of plaque neovascularization in vulnerable or stable plaques.

## Supporting Information

Figure S1 **Data for enhanced intensity (EI) measurements and intra-observer agreement.** a: Scatter plot of EI measurements shows data for second time (x-axis) and first time (y-axis) measurement of observer 1; line of perfect agreement is shown. b: Agreement plot for EI measurements made by observer 1. The difference between two measurements and mean measurements are plotted. Top and bottom lines show the 95% limits of agreement; middle line shows the mean difference.
(TIF)

Figure S2 **Data for enhanced intensity (EI) measurements and inter-observer agreement.** a: Scatter plot of EI measurements (s) shows data for observer 2 (x-axis) and observer 1 (y-axis); line of perfect agreement is shown. b: Inter-observer agreement plot for EI measurements made by observers 1 and 2. The difference between the observers' measurements and the mean measurements are plotted. Top and bottom lines show the 95% limits of agreement; middle line shows the mean difference.
(TIF)
