# Supplementary figures and images for: Relationship between Enhanced Intensity of Contrast Enhanced Ultrasound and Microvessel Density of Aortic Atherosclerostic Plaque in Rabbit Model
Source: PLoS One. 2014 Apr 8;9(4):e92445. doi: 10.1371/journal.pone.0092445 (PMC3979663; doi:10.1371/journal.pone.0092445)

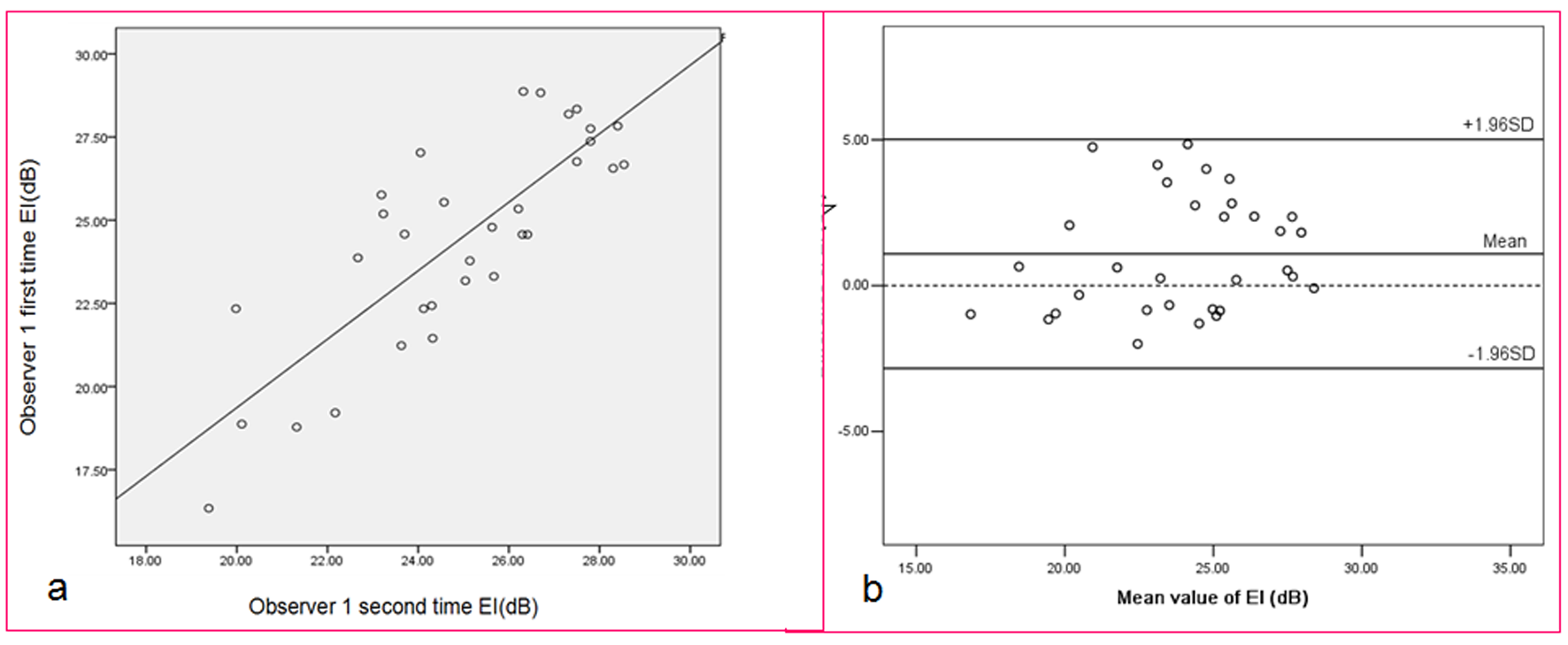

Supplement: Figure S1 — Data for enhanced intensity (EI) measurements and intra-observer agreement. a: Scatter plot of EI measurements shows data for second time (x-axis) and first time (y-axis) measurement of observer 1; line of perfect agreement is shown. b: Agreement plot for EI measurements made by observer 1. The difference between two measurements and mean measurements are plotted. Top and bottom lines show the 95% limits of agreement; middle line shows the mean difference. (TIF) [file pone.0092445.s001.tif]

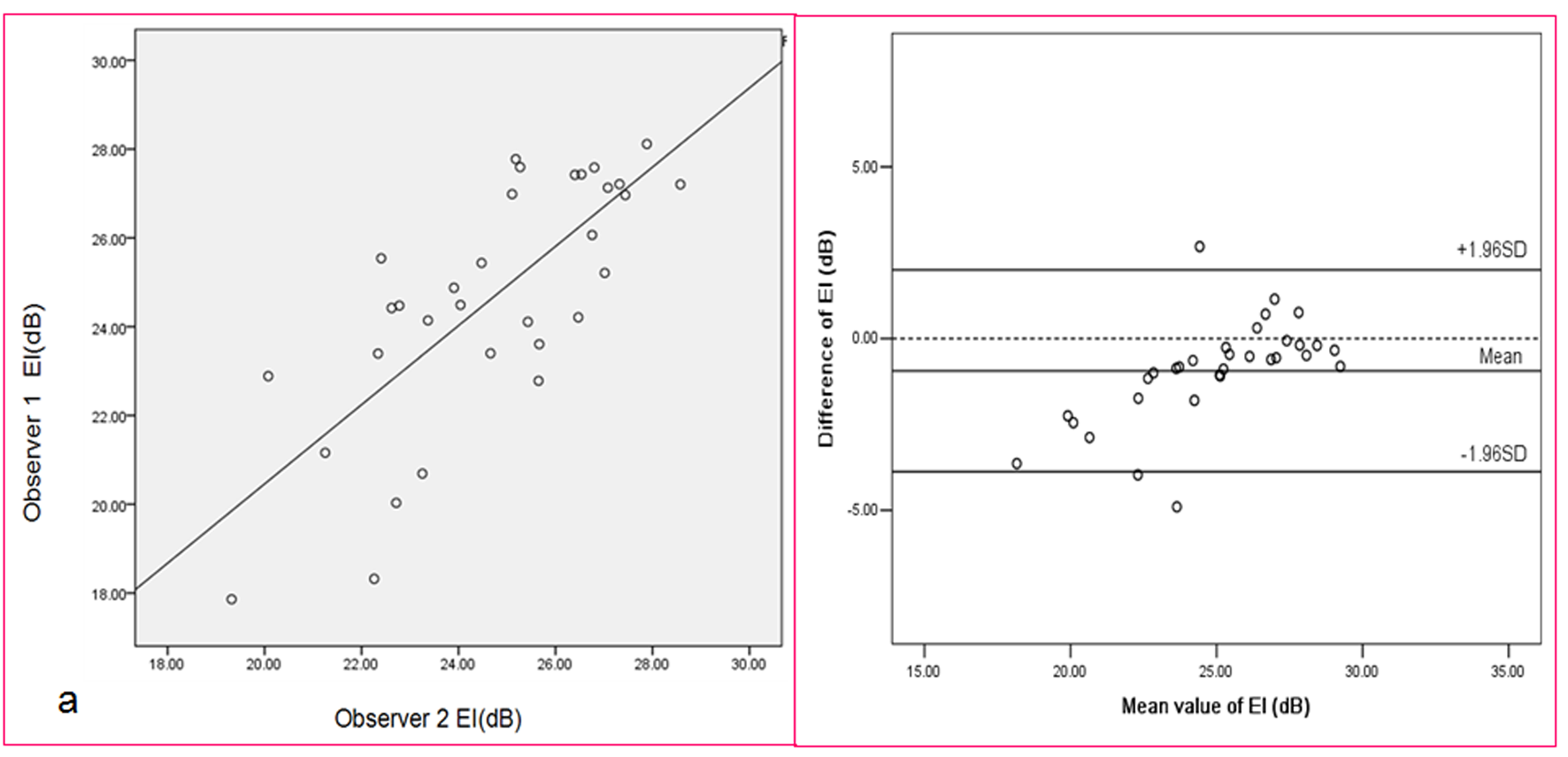

Supplement: Figure S2 — Data for enhanced intensity (EI) measurements and inter-observer agreement. a: Scatter plot of EI measurements (s) shows data for observer 2 (x-axis) and observer 1 (y-axis); line of perfect agreement is shown. b: Inter-observer agreement plot for EI measurements made by observers 1 and 2. The difference between the observers' measurements and the mean measurements are plotted. Top and bottom lines show the 95% limits of agreement; middle line shows the mean difference. (TIF) [file pone.0092445.s002.tif]
